# Designing combination therapies with modeling chaperoned machine learning

**DOI:** 10.1371/journal.pcbi.1007158

**Published:** 2019-09-09

**Authors:** Yin Zhang, Julie M. Huynh, Guan-Sheng Liu, Richard Ballweg, Kayenat S. Aryeh, Andrew L. Paek, Tongli Zhang

**Affiliations:** 1 Division of Biostatistics and Epidemiology, Cincinnati Children’s Hospital Medical Center, Cincinnati, Ohio, United States of America; 2 Molecular and Cellular Biology, University of Arizona, Tucson, United States of America; 3 Department of Pharmacology and Systems Physiology, College of Medicine, University of Cincinnati, Cincinnati, Ohio, United States of America; Duke University, UNITED STATES

## Abstract

Chemotherapy resistance is a major challenge to the effective treatment of cancer. Thus, a systematic pipeline for the efficient identification of effective combination treatments could bring huge biomedical benefit. In order to facilitate rational design of combination therapies, we developed a comprehensive computational model that incorporates the available biological knowledge and relevant experimental data on the life-and-death response of individual cancer cells to cisplatin or cisplatin combined with the TNF-related apoptosis-inducing ligand (TRAIL). The model’s predictions, that a combination treatment of cisplatin and TRAIL would enhance cancer cell death and exhibit a “two-wave killing” temporal pattern, was validated by measuring the dynamics of p53 accumulation, cell fate, and cell death in single cells. The validated model was then subjected to a systematic analysis with an ensemble of diverse machine learning methods. Though each method is characterized by a different algorithm, they collectively identified several molecular players that can sensitize tumor cells to cisplatin-induced apoptosis (sensitizers). The identified sensitizers are consistent with previous experimental observations. Overall, we have illustrated that machine learning analysis of an experimentally validated mechanistic model can convert our available knowledge into the identity of biologically meaningful sensitizers. This knowledge can then be leveraged to design treatment strategies that could improve the efficacy of chemotherapy.

## Author summary

Combination chemotherapy is frequently used in the fight against cancer as treatment with multiple chemotherapy drugs of different molecular mechanisms reduces the chance of resistance. The complex mechanisms involved makes it essential to develop a comprehensive computational model that comprehends experimental data and biological knowledge to facilitate design of combination therapies. As computational models grow and capture more and more molecular events governing the chemotherapy response, it becomes harder to explore the treatment space efficiently and systematically. To facilitate the extraction of unbiased solutions from complicated models, we have conducted systematic analysis using a series of machine learning methods including Partial Least Squares regression (PLS), Random forest (RF), Logistic Regression (LR) and Support Vector Machine (SVM). The results of these different methods were cross-validated to reduce the chance of overfitting or bias by any single method. Overall, we propose a novel computational pipeline, where machine learning analysis of experimentally validated models is used to generate unbiased predictions of novel chemotherapy targets.

## Introduction

Though chemotherapy is one of the most successful tools in the fight against cancer [1], treatment often fails due to a diverse set of resistance mechanisms occurring in cells [2–5]. To minimize the probability of resistance, patients are typically treated with multiple chemotherapy drugs with separate molecular mechanisms. The theoretical justifications are that resistance to one drug will not confer resistance to others and the occurrence of cells harboring multiple resistance mechanisms are rare.

Although better than any single drug, combination chemotherapy presents its own set of challenges, since the drugs interact in complex ways and in some cases might even antagonize one another. Moreover, individual drugs elicit responses on a wide range of timescales, therefore dosing schedules can have a profound effect on the response. In extreme cases, the interaction between drugs can be inverted; that is, one dose schedule can be synergistic while another is antagonistic [6] Given the large number of chemotherapy drugs available, and the almost limitless possibilities for dose schedules, identifying the optimal treatment protocols with experiments alone would be too time and resource consuming to be feasible.

Computational models, which capture the key molecular events of the chemotherapy response, could drastically facilitate the identification of optimal treatment protocols as they can explore treatment space much more rapidly than experimental methods. To this end, we employed a computational modeling strategy to understand the response of HCT116 colon cancer cells at the single cell level to two drugs, the cross linking agent cisplatin and the TNF-related apoptosis-inducing ligand (TRAIL). These drugs induce cell death through two very different mechanisms. Cisplatin induces DNA damage that stabilizes the transcription factor p53 and ultimately leads to activation of the intrinsic apoptosis pathway. In contrast, TRAIL is a cytokine that induces the extrinsic apoptosis pathway by binding to DR4/5 receptors and activating caspase 8 through a p53 independent mechanism [7–9]. Importantly, quantitative single-cell data exists for the response to each treatments making them ideal modeling candidates as the models can be tested to ensure they match the data [5, 10].

To accurately model the response of these two drugs, we combined our previous models on p53 signaling [11] and the intrinsic apoptosis pathway [12] together with recent studies on how intercellular variability contribute to cell fate in response to pro-apoptosis signals. In particular, a recent study by Márquez-Jurado *et al*. revealed that cell-to-cell heterogeneity in mitochondrial mass results in different levels of pro- and anti- apoptotic regulating proteins (e.g. BCL family member MCL, BH3 family member Bid, and the caspase family proteins) and fine-tunes the apoptotic responses of individual tumor cells [13]. Based on these findings, we incorporated variability in apoptosis regulators (BCL family Bax and BH3 proteins) as well as variability in caspases.

We first validated this expanded model by testing predictions on the cellular response to a cisplatin and TRAIL combination treatment. Our model predicts that the combination with TRAIL considerably increases the rate of cell death induced by cisplatin treatment, which we experimentally verified using population measurements. In addition, the model suggests the combination cisplatin/TRAIL treatment leads to a bimodal distribution in the time of death for individual tumor cells. We experimentally confirmed this prediction using live single-cell time lapse microscopy. Moreover, the precisely determined timing of the 2^nd^ wave of death shown by microscopy allows us to refine the decay rate of TRAIL in the model. Such consistency between model prediction and experimental validation suggests that the expanded model serves as a reasonable framework to connect observed cellular fates to available knowledge on the molecular control network.

We further analyzed how cell-to-cell differences in other regulators in the molecular control network (e.g. p53, Bcl-2 and caspase family proteins) impact the cellular response (survival or death) to cisplatin treatment. Due to the large number of components, our validated model is too complicated to yield an analytical solution. Therefore, to gain insight into this complex process, we subjected a heterogeneous population of models that represent individual tumor cells to systematic analyses using an ensemble of machine learning methods. These methods included Partial Least Squares regression (PLS), Random Forest (RF), Logistic Regression (LR) and Support Vector Machine (SVM). The results of these different methods were cross-compared to reduce the chance of overfitting as well as potential bias induced by any single method.

Collectively, these analyses revealed that the fate of cisplatin treated cells are most sensitive to the levels of the classical apoptosis inhibitor BCL family proteins, the pro-apoptotic protein Bax, the positive feedback controlling p53 activation, and the degradation rate of the Bcl-2 homology domain 3 (BH3) proteins, among other regulators. These results are consistent with available knowledge of the biological system. Indeed, the role of Bcl-2 in fine-tuning fates of cisplatin treated cells has been experimentally demonstrated in previous studies [10]. These biologically meaningful results, achieved from machine learning analysis of the solid, experimentally validated models, provided a ‘proof of principle’ of our novel pipeline to integrate knowledge and data to cope with uncertainties and design novel treatments.

Overall, we have successfully integrated high resolution experimental data and currently available knowledge into a validated and comprehensive model that connects the non-genetic variations within cells to their fates in response to combination chemotherapy treatment. The model then represents our knowledge in a suitable framework for machine learning methods to make quantitative discoveries on chemotherapy sensitizers. Furthermore, the model allows us to extract causal insights on how the sensitizers work. Our innovative pipeline, in which mechanistic modeling chaperones machine learning, is empowered by the strength of these two different approaches, and could provide a way to efficiently identify effective drug combinations for a diverse set of cancers.

## Results

### An expanded model that incorporates multiple sources of variability

**Fig 1:**
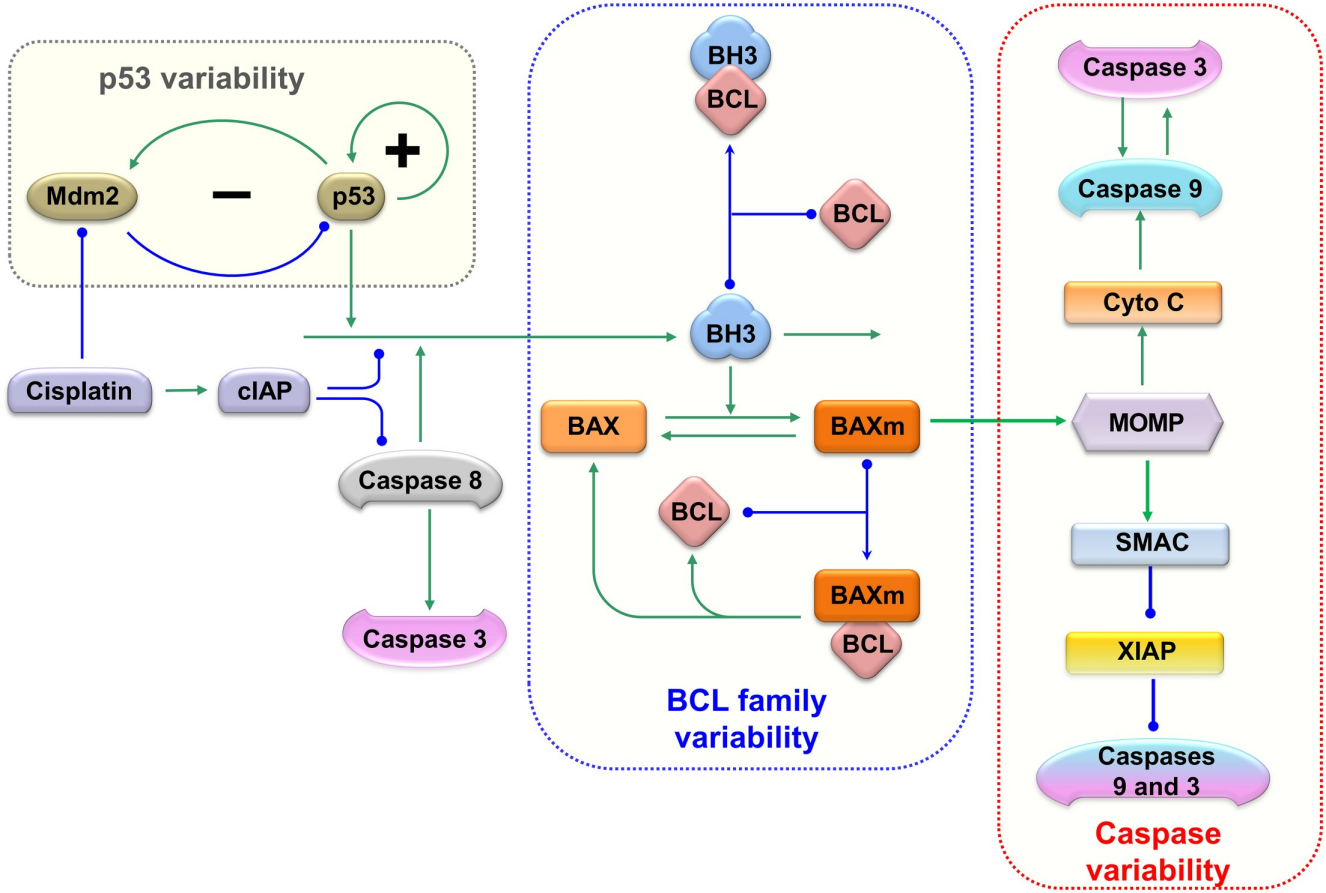
The molecular interactions and variabilities described in the current model. Nodes of different shapes represent the model components; arrows indicate activation; lines and curves with solid circle heads indicate repression. The model contains four sources of variability arising from p53, caspase 8 activation, BCL-family proteins and Caspase proteins. The variability of p53 (highlighted in grey rectangle) is inherited from our previous model [46]. Meanwhile, caspase 8 is activated with different rates; the expression of BCL family proteins (in blue dashed rectangle); and the caspase proteins (in red rectangle) are also varied between individual cells.

The current model for fractional killing incorporates three sources of variability: p53 regulation, BCL family proteins, and caspase proteins (Fig 1). Heterogeneity in the apoptosis regulator BCL proteins as well as caspases was incorporated due to recent work by Márquez-Jurado *et al*. [13], who showed that variation in the pro- and anti- apoptotic proteins Bcl-2, Bax, Smac, XIAP, Caspase-8 and Caspase-9 fine-tuned the apoptotic responses among individual cells [13].

**Fig 2:**
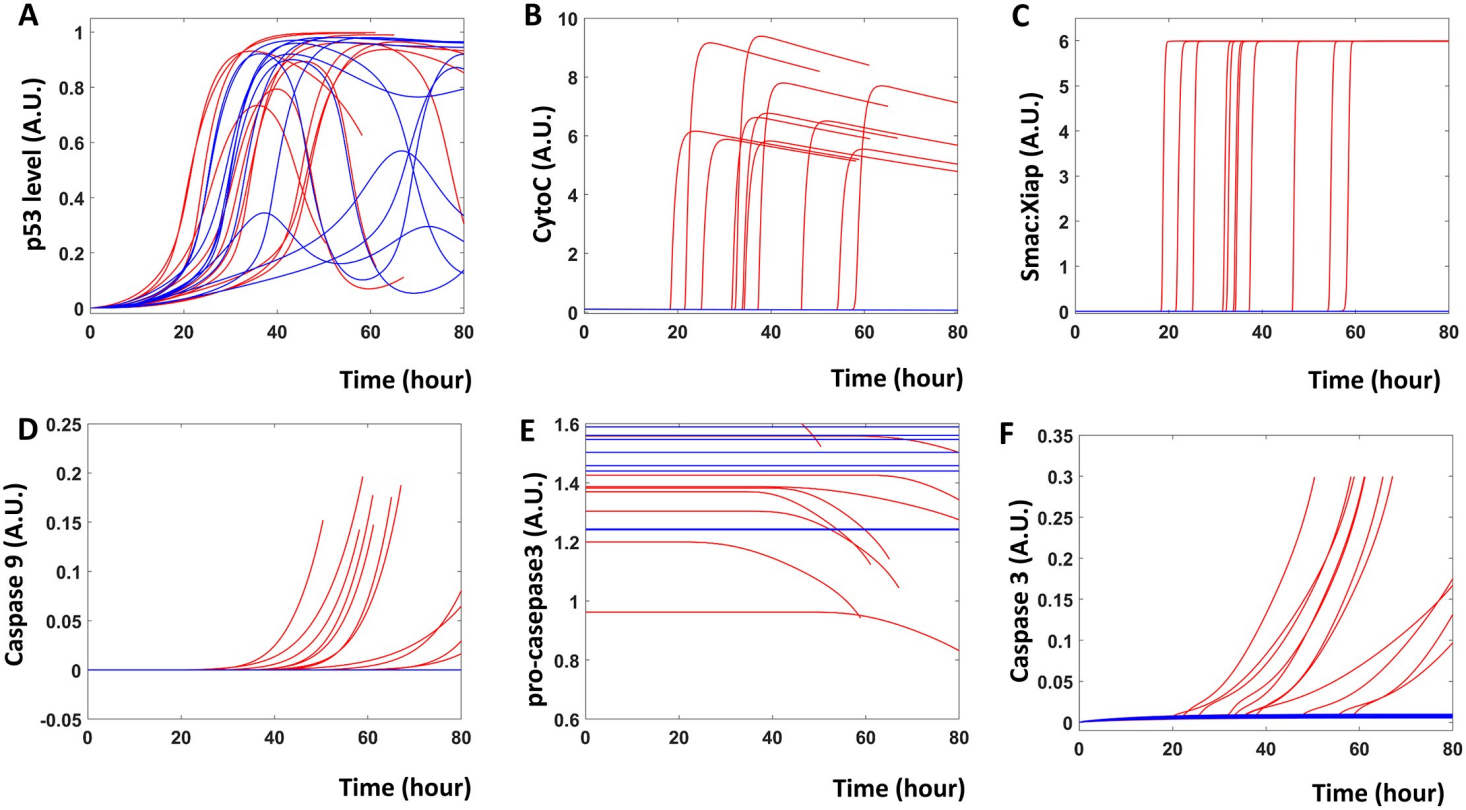
The current model describes the sequence of events during fractional killing. Time series simulation of the current model provides a comprehensive picture of the molecular events during cisplatin induced fractional killing. Blue traces indicate cells that survived treatment, while red traces indicate cells that underwent apoptosis (40.6% of total). Cisplatin treatment results in the activation of p53 (**A**), the release of CytoC from mitochondria (**B**), the inactive dimer of Smac and XIAP (**C**). Eventually, cisplatin leads to the activation of caspase 9 (**D**), cleavage of pro-caspase 3 (**E**) and the activation of caspase 3 (**F**).

The influence diagram (Fig 1) of the expanded model was translated into a set of ordinary differential equations (ODEs, details in Methods). Holding these equations constant, we varied equation parameters to simulate a population of models each representing a single cell, thus mimicking a population of tumor cells with cell-to-cell variability. The temporal simulations of representative cells are shown in Fig 2. In response to an identical concentration of cisplatin, some cells died (red curves, Fig 2), while others survived (blue curves, Fig 2). Cells with different fates activate p53 at different rates, with dead ones activating p53 faster (Fig 2A), matching previously published experimental results [10]. In apoptotic cells, rapidly accumulating p53 activates Baxm proteins, which results in the release of CytoC from mitochondria (Fig 2B). Meanwhile, Smac is also released from mitochondria and the apoptosis inhibitor XIAP is sequestered into the inactivated Smac:XIAP dimer form (Fig 2C). Together, CytoC and Smac result in the activation of caspase-9 (Fig 2D), which cleaves inactive pro-caspase-3 into the activated form of caspase-3 (Fig 2E and 2F). Once activated, caspase-3 can directly induce cell death [5, 12]. Cell death is triggered and simulation stops when caspase 3 activity rises above a threshold level of 0.3.

### Experimental validation of the expanded model

**Fig 3:**
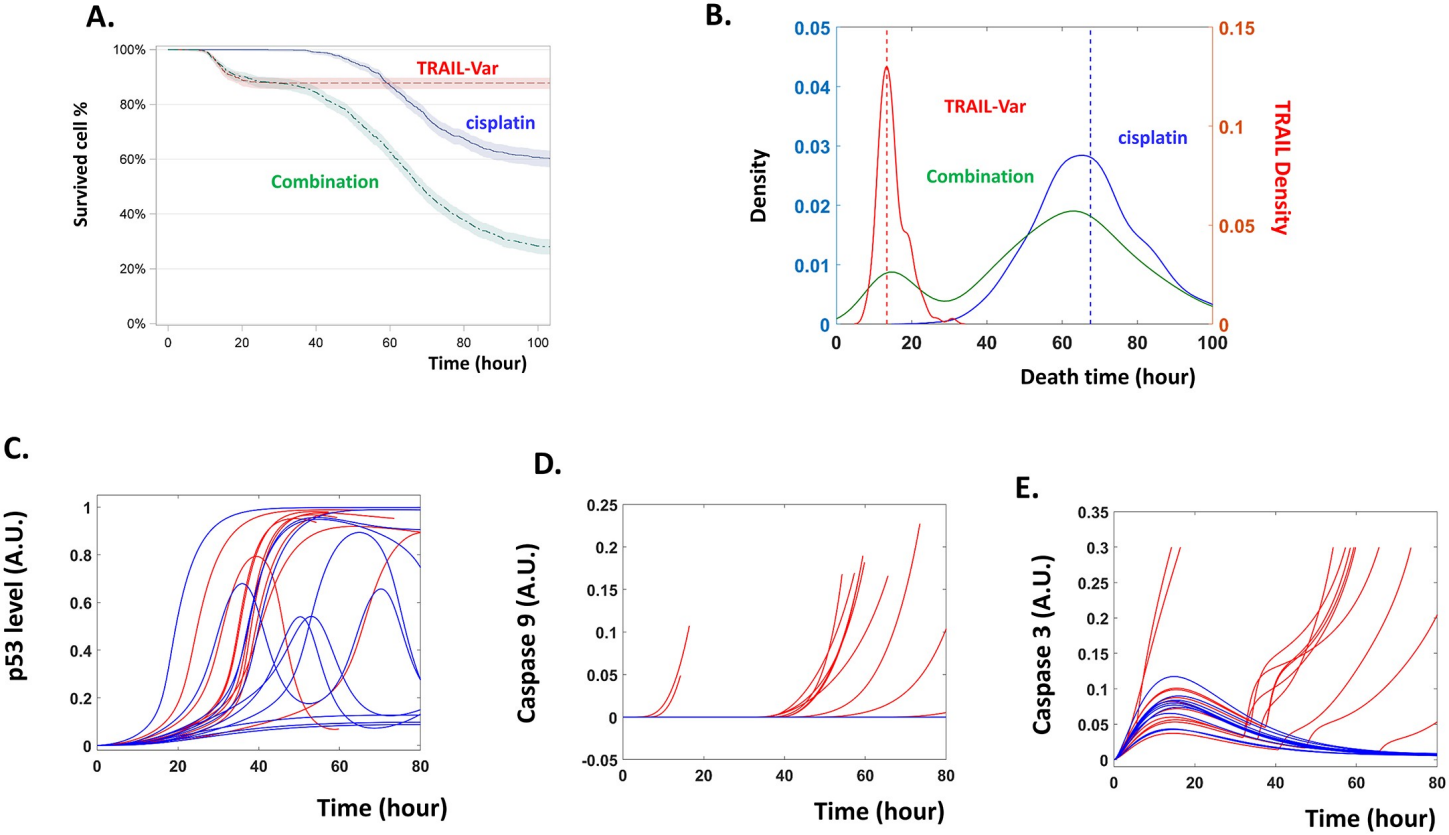
Simulated dynamics that characterize the combined therapy. (**A**). Kaplan-Meier plot of the simulated data with TRAIL functioning as a dynamical variable. (**B**). Density plots of the distribution of the time of death predicted by the model for TRAIL alone (right y-axis), cisplatin or cisplatin+TRAIL combination (left y-axis). TRAIL functions as a dynamical variable. (**C-E**). Temporal simulation of p53 (C), caspase-9 (D) and caspase-3 (E) following cisplatin+TRAIL combination.

We next validated this expanded model by experimentally examining its predictions. Cancer cells also undergo fractional killing in response to treatment with TRAIL ligand [5], and our model predicts that the incorporation of TRAIL would enhance the killing efficiency of cisplatin (Fig 3A and 3B). Kaplan-Meier plotting suggests that the combined therapy results in more rapid death as well as a higher cellular mortality rate. Cisplatin alone induces the death of 40.6% of treated cells, while the combination of cisplatin with TRAIL increases the rate of cellular death to around 73.7% (Fig 3A). All other settings in these two populations are identical.

Furthermore, density plots of the distribution of death times suggest that the cisplatin and TRAIL combination induces “*two waves of death”*: the initial peak is around 18 hours after treatment and corresponds to the peak time observed with TRAIL alone; and the 2^nd^ peak time is around 65 hours, similar to the single peak induced by cisplatin alone (Fig 3B). From the temporal simulations following combination therapy, we could see that as the p53 dynamics changes little in response to the combination therapy, the combination results in two waves of activations of Caspase 9 and Caspase 3 (Fig 3C–3E).

This predicted ‘*two waves of death*’ indicates that the level of TRAIL ligand does not remain constant but decays over time, as assumed in the current model. Indeed, if an alternative assumption is made and the level of TRAIL is held constant, the combined therapy results in only one large wave of cellular death (S1A and S1B Fig).

Although the timing of cell death is sensitive to the TRAIL decay rate, the prediction of enhanced cellular death is robust and insensitive to it; a higher fraction of cells will die in response to the combination treatment, whether TRAIL decays or remains constant (Fig 3A and S1A Fig). Hence, the model generates two predictions, one robust prediction of higher death fraction and one less robust prediction of ‘two waves of death’ (Fig 3).

**Fig 4:**
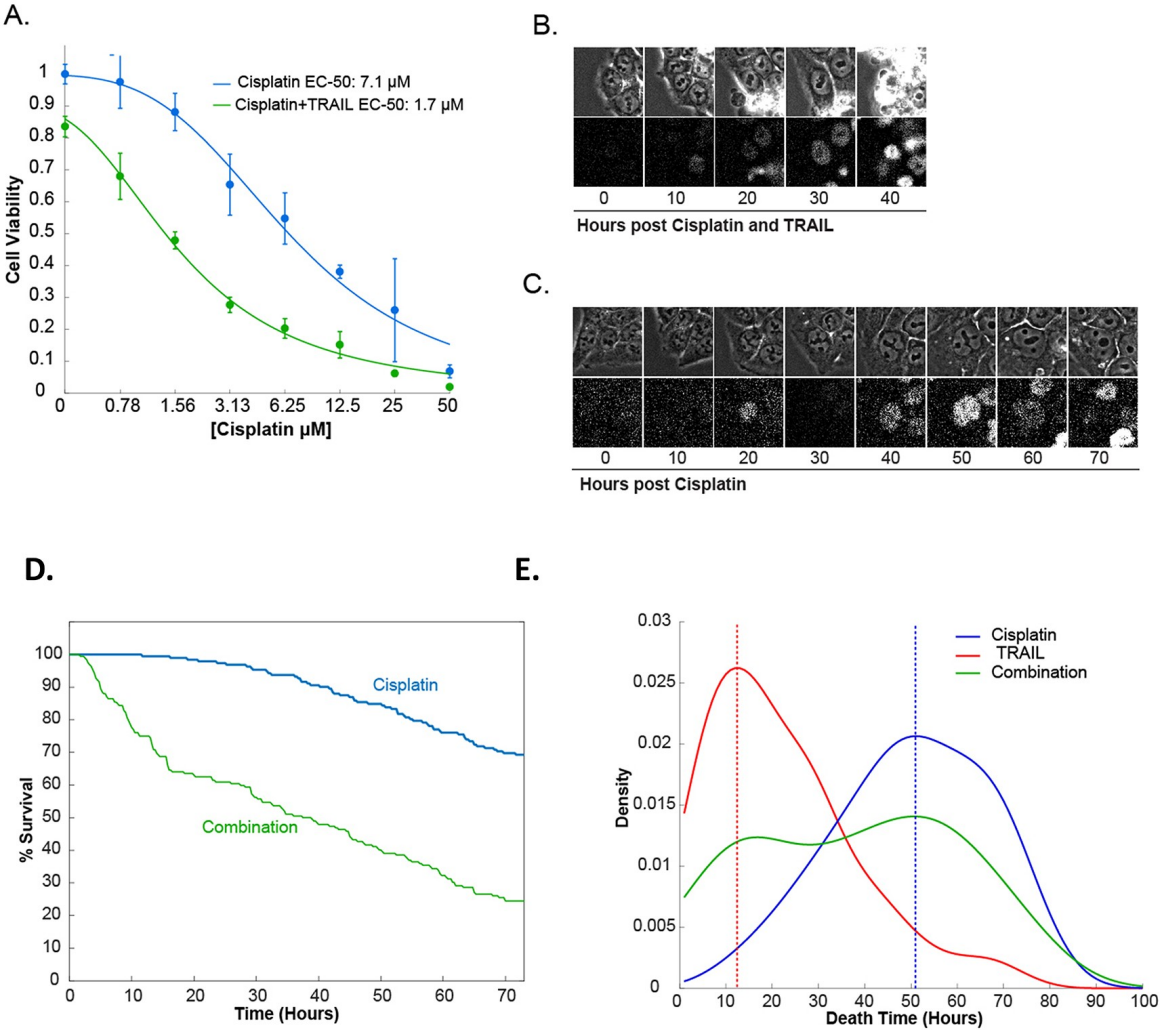
Experimental data for the combination therapy with TRAIL and cisplatin. Cultured HCT116 cells were subjected to treatments with cisplatin alone, TRAIL alone, or cisplatin plus TRAIL, respectively. (**A**) Dose-response curve of HCT116 cells 72 hours after treatment with different concentrations of cisplatin, in the absence (blue) or presence (green) of 25 ng/ml of TRAIL. Error bars represent the standard deviation of four biological replicates. (**B** and **C**) p53-Venus dynamics were measured by live-cell microscopy (lower panel), and cell death was identified by morphological changes of the cells measured by phase contrast microscopy (top Panel, see middle cell in C at 40 hours for example of an apoptotic cell). (**D**). A Kaplan-Meier plot of the experimental data. 192 single cells were tracked for each dose. (**E**) Density plot of the distribution of the time of death measured in live-cell microscopy time-lapse experiments. Dashed red line represents the peak time for cell death for TRAIL; dashed blue line is the peak time of death for cisplatin.

We proceeded to test these model predictions of death time and death rate. We first generated dose-response curves for HCT116 colon cancer cells treated with cisplatin alone or a combination of cisplatin with a low dose of TRAIL (Fig 4A). Consistent with the model’s robust prediction, the combination treatment led to a decrease in cell viability when compared to cisplatin alone (Fig 4A, cisplatin EC_50_ 7.1 μM **V.S**. cisplatin + TRAIL EC_50_ 1.7 μM).

The population measurements generated by the dose-response curves cannot detect the dynamical features of the cells, such as time to death. Testing whether there are ‘*two waves of death*’ requires observing death events in single cells with high temporal resolution. Therefore, we used time-lapse microscopy to track the kinetics of cisplatin and TRAIL induced cell death over time. We used a previously engineered colon cancer cell line where one allele of p53 was tagged with the Venus fluorescent protein at the endogenous locus and harbored mCerulean fused to a nuclear localization signal to track nuclear p53 levels (HCT116 p53-VKI, [10, 14]). In these single cells, p53-Venus levels and cell fate (death or survival) were measured following the treatments with cisplatin alone and cisplatin + TRAIL by time-lapse fluorescence microscopy every 15 minutes over a 74 hour period (Fig 4B and 4C, S1–S3 Movies). Kaplan-Meier plotting revealed that the combination TRAIL plus cisplatin treatment killed more cells and at a faster rate when compared to cisplatin treatment alone (Fig 4D). Furthermore, the experimental data revealed two waves of death in the cisplatin + TRAIL combination treatment (Fig 4E). Time-lapse images were also acquired for the TRAIL mono treatment, however due to the large number of divisions we were unable to track single cells when they receive only TRAIL. We did measure the times of death of single cells to get the death distributions in Fig 4E. The first peak of death aligned closely with the peak observed with TRAIL treatment alone, while the second peak of death aligned to the cisplatin only therapy. Hence, these data confirm the model assumption that TRAIL actively decays rather than remaining at its initial concentration. The experimental confirmation of the model’s predictions show that the model assumptions are reasonable and indicates that our expanded model can serve as a faithful ‘*in silico*’ representation of the biological system.

### Systematic machine learning identifies chemotherapy sensitizers

Since the expanded model incorporates the relevant data into a suitable, knowledge constrained framework, we hypothesized that rigorous analysis of this model could yield biologically meaningful results. To test this hypothesis, the expanded model was simulated with a total of 6000 different sets of parameters whose values were randomly selected (details in Methods). In this way, these simulations served as an “*in silico”* proxy of 6000 cisplatin treated tumor cells and are referred to as *in silico cells* (ISCs) hereafter.

**Fig 5:**
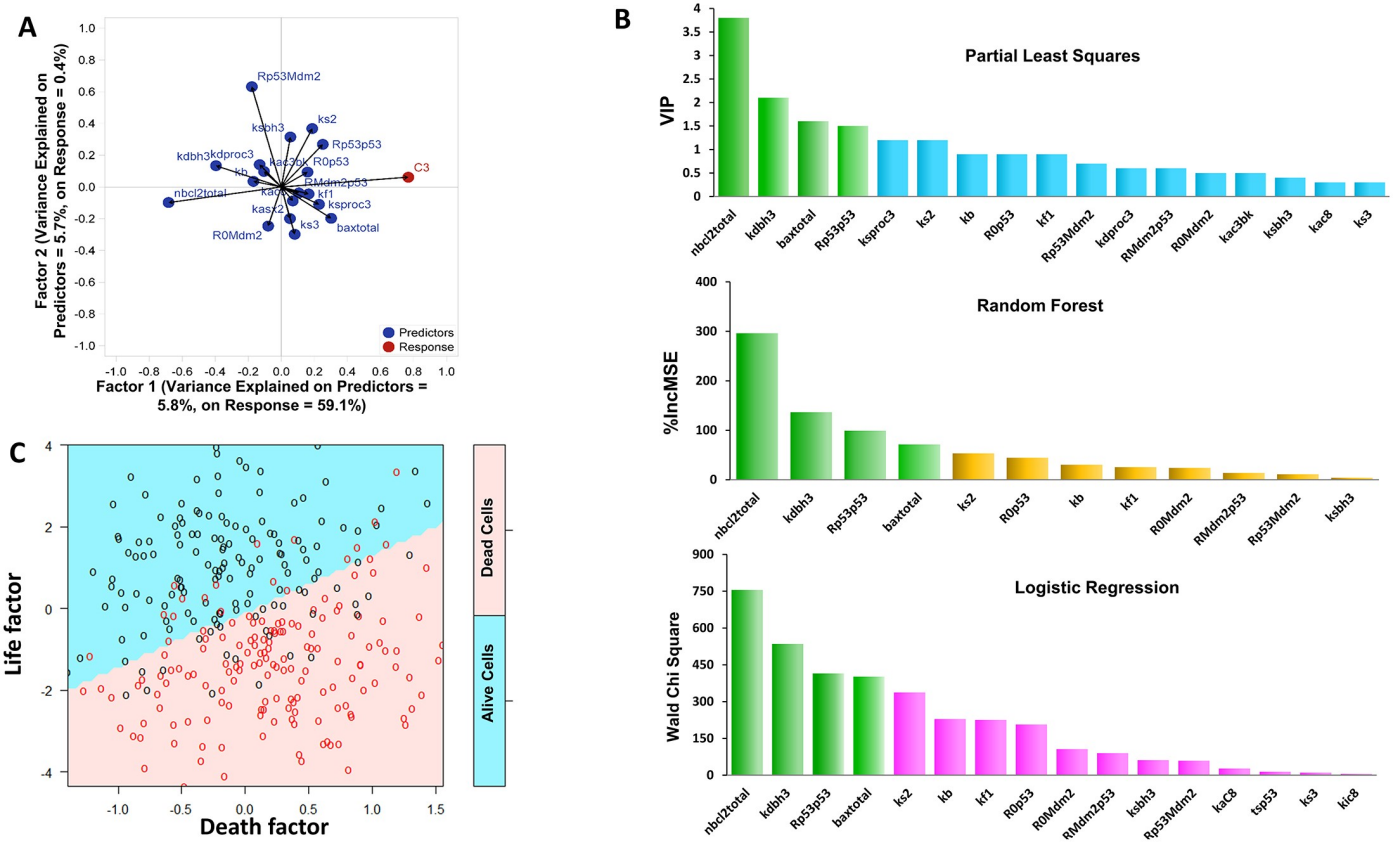
Machine learning analysis of in silico cells. **(A)**, Biplot from PLS analysis, with variances from both parameters and Caspase 3 level explained (**B**), the four components in green are the common ones identified by all these methods. Upper panel: the Variable Importance in Projection (VIP) of parameters achieved by PLS; Middle panel: a hierarchical order of the control parameters based on the percentage increase in the mean squared errors (%IncMSE) in Random forests; Lower panel: the ranking of parameters selected by the logistic regression on the basis of Wald Chi Square; (**C**), The SVM plotting carried out with the life promoting and death promoting factors. Red circles indicate dead cells, while black circles are surviving cells.

Among the population, an ISC is labelled as “*dead*” if caspase-3 is activated past a specific threshold and as “*alive*” if caspase-3 activity is kept below the threshold. In this way, cell fate is determined by the peak value of caspase-3, which is a continuous response variable. As the level of caspase-3 is a systems level property and controlled by many parameters, Partial Least Squares regression (PLS) was used to reduce the parameter dimension and to identify the parameters that contribute most to the value of this continuous variable [15]. PLS identified two principal factors that together explain about 60% of the variance that characterizes the change of peak caspase-3 level (Fig 5A). Based on their relative contributions to these principal factors, PLS then generated a rank of the contributions by individual parameters (Fig 5B, top panel). With this rank, PLS revealed that caspase-3 activity is most sensitive to variabilities characterizing the level of Bcl-2, the level of Bax, the positive feedback controlling the transcription factor p53, and the stability of BH3.

To avoid bias induced by any single analysis method, we subjected the discrete life-death responses of ISCs to two additional machine learning methods: random forest and logistic regression. For our analysis, the random forest established 2000 decision trees to calculate the relative contribution of each variable in distinguishing living cells from dead ones, while logistic regression identified the dominant factors using backward elimination [15]. Consistent with the results from PLS, these two alternative methods also identified the variability of four components (Bcl-2, Bax, p53 self-activation and BH3 stability) as the dominant factors contributing to cell death in response to cisplatin (Fig 5B, middle and bottom panels).

The sensitizers identified by these machine learning methods are consistent with previous studies. Our previous work has shown that cell death in response to cisplatin can be greatly enhanced by inhibiting BCL proteins [10]. Meanwhile, other studies using either gain-of-function [16–18] or loss-of-function [19–21] of Bax showed its importance in regulating cell death under diverse conditions. This partial confirmation indicates that indeed, machine learning analysis of knowledge based, data constrained mechanistic models can yield biologically meaningful sensitizers that are informed by both experimental data and biological knowledge.

Furthermore, the identified controlling components have opposite effects on cellular death. As the elevation of p53 positive feedback strength and Bax level promote cellular death; the increase of the other two components (BCL level and BH3 stability) promote cellular survival. On the basis of these biological considerations, we then carried out support vector machine (SVM) analysis with a linear combination of the two life promoting components (Fig 5C, y-axis) and the two death promoting components (Fig 5C, x-axis). The combination coefficients were identified using independent logistic regressions (details in Method). As a result, SVM plotting identified a two dimensional plane with two clearly distinguished regions: the top left region is mainly occupied by living cells, while the bottom right region contains mostly dead cells (Fig 5C).

Starting from a high dimensional space of over 30 parameters, this SVM identified a 2-dimentional plane that correctly identified the fates of a majority of cells (84.5%). In this way, the SVM identified boundary derived from simulated data provides an elegant way to simplify and to understand the complex control of cellular fates.

## Discussion

Mechanistic modeling has been widely used to aid in the design of effective cancer therapies or to identify biomarkers for personalized treatment [22–35]. Recently, machine learning methods have also gained popularity in multiple areas including biomedicine [36–38]. Given their distinct power and limitations, it is reasonable to expect that the integration of these two different methods could result in a powerful tool for the biomedical community [39]. Indeed, Gong and Sobie have elegantly integrated mechanistic modeling and machine learning to predict drug responses across different types of cultured cells [40].

In this work, we illustrate a novel way to use mechanistic modeling to chaperone and improve machine learning to extract biologically meaningful results. By incorporating existing knowledge and providing mechanistic explanations, mechanistic modeling allows us to cope with two major limitations of machine learning performed in isolation: being unable to incorporate available knowledge and lack of causality. Hence, this novel, integrated approach utilizes the power of machine learning and reduces its limitations.

Recently, machine learning analysis has gained rapid popularity and there is concern on how the biomedical community should apply this new tool in conjunction with traditional mechanistic modeling [39, 41, 42]. In this exploration, we revealed one plausible way to integrate these two, in which the machine learning algorithms were used to shed light on the control of the mechanistic model when all its parameters are changed simultaneously. In doing so, we achieved a robust and consistent ranking of the model components, as well as a clearer representation of the system on the reduced two dimensional SVM plane. By efficiently utilizing these methodologies, we believe that the traditional field of mechanistic modeling can benefit from the rapid development of machine learning algorithms.

Meanwhile, our work also indicates that solid and well-validated models are essential for this integrated approach to be successful. We believe that mechanistic models can only become solid if they are continuously validated and improved against experimental findings, and we have insisted in continuously testing and modifying our model with novel experimental results.

**Fig 6:**
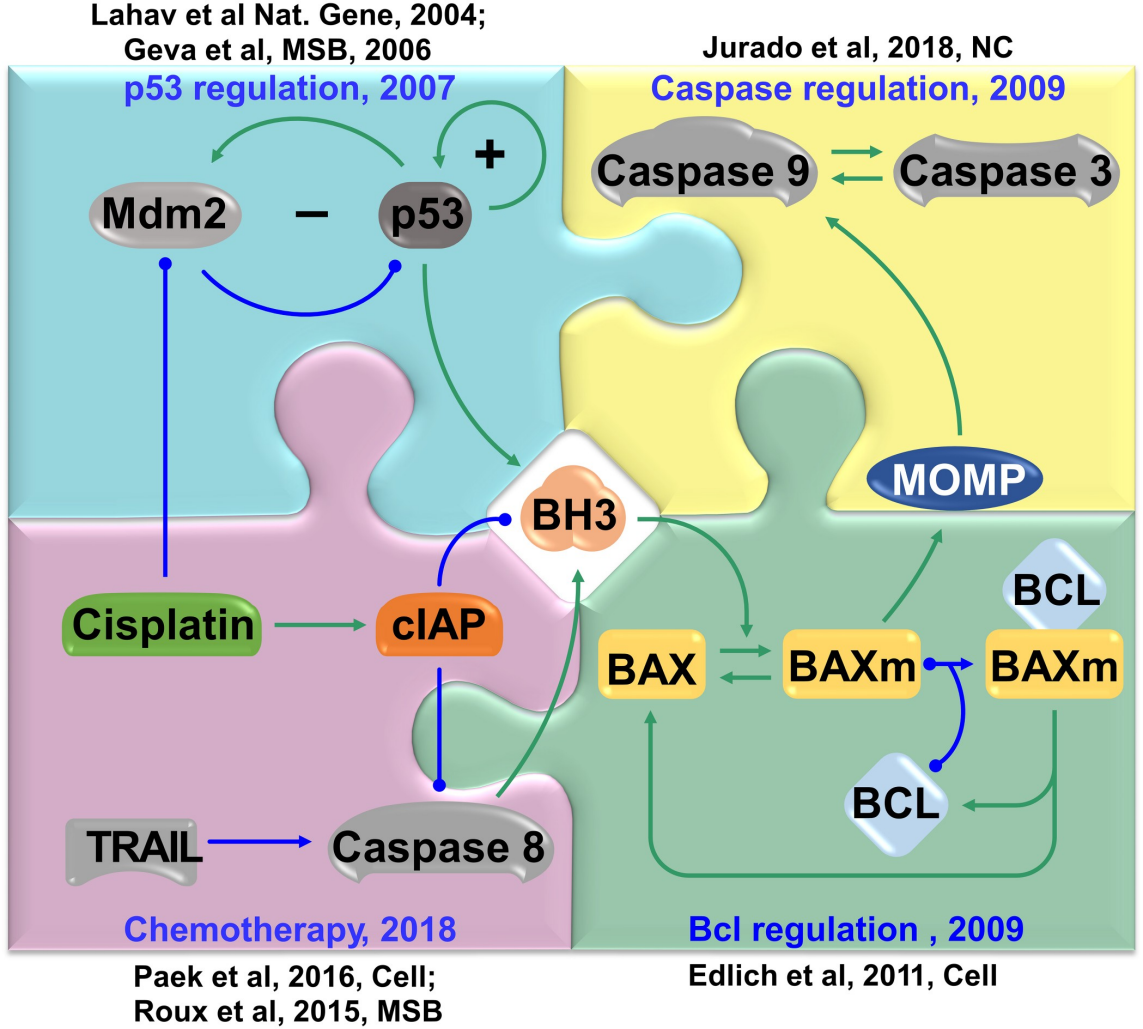
Interplay between model and experimental observation. The outer pieces show the experimental work that were used for the construction, verification, expansion, and refinement of the models. The inner pieces indicate the pieces of the model.

A brief time line of the model’s history and its interaction with relevant experimental studies is shown in Fig 6. Initiated by dynamical studies on p53 oscillations [43, 44], our original model for the pulsatile dynamics of p53 in 2007 [11]. Meanwhile in 2009, we also developed a computational framework for the intrinsic cell death pathway [12].

At the time, the model of programmed cell death made a novel assumption. It assumed that the inactivation of the mitochondrial form of Baxm is catalyzed by the apoptosis inhibitor BCL family proteins. In other words, BCL family proteins serve not only as stoichiometric inhibitors, but also as enzymes to accelerate the inactivation of Baxm from the mitochondria and shuttle them back to the cytoplasm. This essential model assumption was then validated independently by Edlich *et al*. [45]. By observing fluorescence loss in photobleaching, Edlich *et al*. demonstrated that the translocation of Bax from the mitochondria to the cytoplasm is sped up when a member of the BCL family, Bcl-xl, is overexpressed; on the contrary, this translocation rate decreased when Bcl-xl is inhibited, as reported in Fig 6D from [45]. This independent confirmation of the model assumption suggested that the model is reasonable and can be used for further expansion and predictions.

In 2016, the high temporal resolution data reported by Paek, *et al*. suggested an intriguing relationship between the dynamical activation of p53 and cellular fates [10]. Following this report, we developed a comprehensive model that combined our models of p53 and apoptosis while incorporating the newly found incoherent pathways activated by the chemotherapy drug cisplatin. The model successfully recaptured and explained the observed dynamic relation [46]. In the current study, we further expanded the available model with recent data from the literature and from the Paek lab. When additional data is available in the future, the model will be further improved to better represent our growing knowledge of the biological system.

Since our mechanistic model has been continuously updated and validated with experimental data from multiple sources, it currently serves as a general apoptosis framework. Because each component of the combined model was derived from models tested on different cell types, it yields results that broadly apply to many different cell types. We hope that in the near future, the apoptosis model can be constrained with data from a single cell line. Then, integrating the cell line specific model with machine learning analysis will promise to yield novel, specific results in that particular cell line. Meanwhile, the methodology we apply in the manuscript integrates the powers of both mechanistic modeling and machine learning, and achieves more than either approach in isolation. This approach can be generally applied to study a broad range of cancers as well as other complex diseases.

## Methods

### Simulating a population of cells with different fate

The wiring diagram of the model was translated into a set of ordinary differential equations (S1 Table) and simulated following our established protocol [46]. The structure and basal parameter values of the model are mostly inherited from previous publications [11, 12, 46]. The rate constants are characterized by the inverse of the time scale of model (hour ^-1^). The levels of the control molecules, as well as the regulatory strengths, are dimensionless. For the population level simulations, the parameters were randomly sampled from uniform distributions ranging between 70%–120% of their basal levels, which were used to properly recapture the corresponding experimental observations from our previous publication [10, 46]. The models are simulated with stiff methodology in the software Xppaut (http://www.math.pitt.edu/~bard/xpp/xpp.html). The simulated living cells and death cells in response to different drug treatments, these simulated cells are then subject to analysis with machine learning methods.

### Machine learning analysis of the simulated data

An ensemble of machine learning methods was used to analyze the model simulated data. Partial Least Square (PLS) Analysis was used for dimension reduction with multivariate linear regression [15]. Similar to Principal Component Analysis (PCA), PLS also identifies a smaller set of factors that are linear combinations of the original parameters. However, in contrast to an unsupervised PCA, PLS is supervised and the factors identified here do not always include all the original parameters. Rather, only part of the original parameters are included in the PLS-identified factors on the basis of the comparison between the observation and statistical prediction. In the factor space with reduced dimensions, the composed linear regression model bears the smallest distance (least squares) between the measured values and the fitted ones. The PLS was carried out with the standard procedure within the statistical software SAS (*SAS Institute Inc*., *Cary*, *NC*, *USA*).

By assembling multiple Decision Trees, Random Forest was used to improve the classification results and generate a rank of how each parameter contributes to the prediction accuracy [15]. Computation was done in R using the Random forests algorithm within the standard package [47]. Default parameters were used along with 10-fold cross-validation.

Logistic regression, which models the logistic transformation of probability of the binary response cell fates [15], was used to rank the contribution of the control parameters as well as to identify the linear combination of the life promoting and death promoting parameters. The logistic regression, together with backward elimination and 10-fold cross validation, was computed in SAS programming environment using the standard method and default parameter setting. For ranking the control parameters, all parameters are incorporated at the beginning; for identifying the linear combinations, the selected parameters were used.

### Support Vector Machine

The Support Vector Machine (SVM) is a popular classification method. With a subset of the models from the population, SVM computes the boundaries that separate different populations of data with maximal margin. The chosen data are referred to as “*support vectors*” since they are used to compute the boundaries. The SVM was carried out within R.

The “life factor” used in SVM is a linear combination of the BCL-2 level and BH3 degradation rate (−17.6132 + 14.5786 * *nbcl*2*total* + 6.9014 * *kdbh*3), while the “death factor” is a linear combination of the strength of p53 positive feedback and the level of Bax (−6.0579 + 1.5398 * *Rp*53*p*53 + 3.3541 * *baxtotal*). These coefficients are identified with logistic regression that best fits the probability of cellular survival with Bcl2 level and Bh3 degradation rate; and then fits the probability of cellular death with the p53 positive feedback strength and Bax level.

### Bifurcation analysis

The one parameter bifurcation and the two parameter bifurcation analysis were carried out with death factor and life factor as control parameters, and the position of the threshold between life and death regions was tracked with the freely available Oscill8 software (http://oscill8.sourceforge.net).

### Dose-response curves

Approximately 3,000 HCT116 cells were plated to each well of a 96 well plate in McCoys 5A + 10% FBS. After 24 hours, cisplatin (Sigma, 1134357) and/or TRAIL (Sigma, T9701) was added to the media. 72 hours after drug treatment, cells were washed 2x by PBS and fixed in PBS containing 3.5% paraformaldehyde for 20 minutes at room temperature. Cells were then permeabilized using PBS containing. 1% Triton X-100 for 1 hour at room temperature and then stained using Cell Tag 700 stain (LiCor 926–41090) which stains the nucleus and cytoplasm of cells. Total cell viability was measured using the 700nm channel of a LiCor Odyssey scanner. Cell viability was normalized to untreated cells. *EC*_*50*_ was estimated by fitting the data using MATLAB to the hill function: $E_{Hill}=1+\frac{E_{\infty }-1}{1+{\left(\frac{{EC}_{50}}{C}\right)}^{H}}$ where C is the concentration of cisplatin, *E*_*∞*_ is the maximum effect, *EC*_*50*_ is the 50% effective concentration and *H* is the hill exponent.

### Measurement of p53 dynamics and determination of apoptosis in single cells

To measure p53 dynamics and cellular death in single human colon cancer derived, HCT116 cells, we used the HCT116 p53-VKI cell line which has one allele of p53 tagged at the C-terminus with the Venus fluorescent protein and mCerulean-NLS as described previously [10, 14]. We have previously shown that the p53-Venus reporter matches endogenous p53 dynamics [10]. Cellular morphology was used to determine whether cells have enacted apoptosis (See Fig 4). For microscopy, ~10,000 cells were plated on poly-d-lysine coated glass bottom dishes (MatTek, P35GC-1.5-14-C) and allowed to attach to plates for 72 hours in McCoy’s media 5A Media with 10% FBS. Media was washed out with PBS and replaced with DMEM FluoroBrite (ThermoFisher) with 5% FBS and 1% Glutamax (ThermoFisher). Cells were imaged on a Nikon Eclipse Ti-E microscope enclosed in an OKO labs incubation chamber to maintain humidity, a temperature of 37°C and 5% CO_2_. Images were captured every 15 minutes for 74 hours. Drugs were added 1.5 hours into the experiment, and mineral oil was added to prevent evaporation of media. We used previously written custom MATLAB scripts to extract p53 dynamics and cell death from single-cells.

## Supporting information

S1 Fig Simulated dynamics that characterize the combined therapy. TRAIL is assumed to remain constant. (**A**). Kaplan-Meier plot of the simulated data with TRAIL functioning as a dynamical variable. (**B**). Density plots of the distribution of the time of death predicted by the model for TRAIL alone (right y-axis), cisplatin or cisplatin+TRAIL combination (left y-axis). TRAIL functions as a dynamical variable.
(PPTX)

S1 Movie HCT116 p53-VKI cells treated with 6.25 μM cisplatin 1.5 hours into the movie. p53-Venus is colored in yellow, scale bar at bottom right, time stamp at top left. Contrast of p53-Venus channel is the same in all three movies.
(MP4)

S2 Movie HCT116 p53-VKI cells treated with 25 ng TRAIL. Cisplatin was added 1.5 hours into the movie, p53-Venus is colored in yellow, scale bar at bottom right, time stamp at top left. Contrast of p53-Venus channel is the same in all three movies.
(MP4)

S3 Movie HCT116 p53-VKI cells treated with 6.25 μM cisplatin and 25 ng TRAIL 1.5 hours into the movie, p53-Venus is colored in yellow, scale bar at bottom right, time stamp at top left. Contrast of p53-Venus channel is the same in all three movies.
(MP4)

S1 Data Description of the format of the data files (S2–S4 Data). (DOCX)

S2 Data Time lapse data of HCT116 p53-VKI cells treated with 6.25 μM cisplatin between frame 5 and 6. Data format is described above in **‘SupportingData’**.
(XLSX)

S3 Data Time lapse data of HCT116 p53-VKI cells treated with 25 ng TRAIL between frame 5 and 6. Data format is described above in **‘SupportingData’**.
(XLSX)

S4 Data Time lapse data of HCT116 p53-VKI cells treated with 6.25 μM cisplatin and 25 ng TRAIL between frame 5 and 6. Data format is described above in **‘SupportingData’**.
(XLSX)

S1 Table Model equations, parameters and initial conditions. (DOCX)
