# Supplementary material for: Designing combination therapies with modeling chaperoned machine learning
Source: PLoS Comput Biol. 2019 Sep 9;15(9):e1007158. doi: 10.1371/journal.pcbi.1007158 (PMC6733436; doi:10.1371/journal.pcbi.1007158)
Supplement: S1 Fig — TRAIL is assumed to remain constant. (A). Kaplan-Meier plot of the simulated data with TRAIL functioning as a dynamical variable. (B). Density plots of the distribution of the time of death predicted by the model for TRAIL alone (right y-axis), cisplatin or cisplatin+TRAIL combination (left y-axis). TRAIL functions as a dynamical variable. (PPTX) [file pcbi.1007158.s001.pptx]

## Slide 1
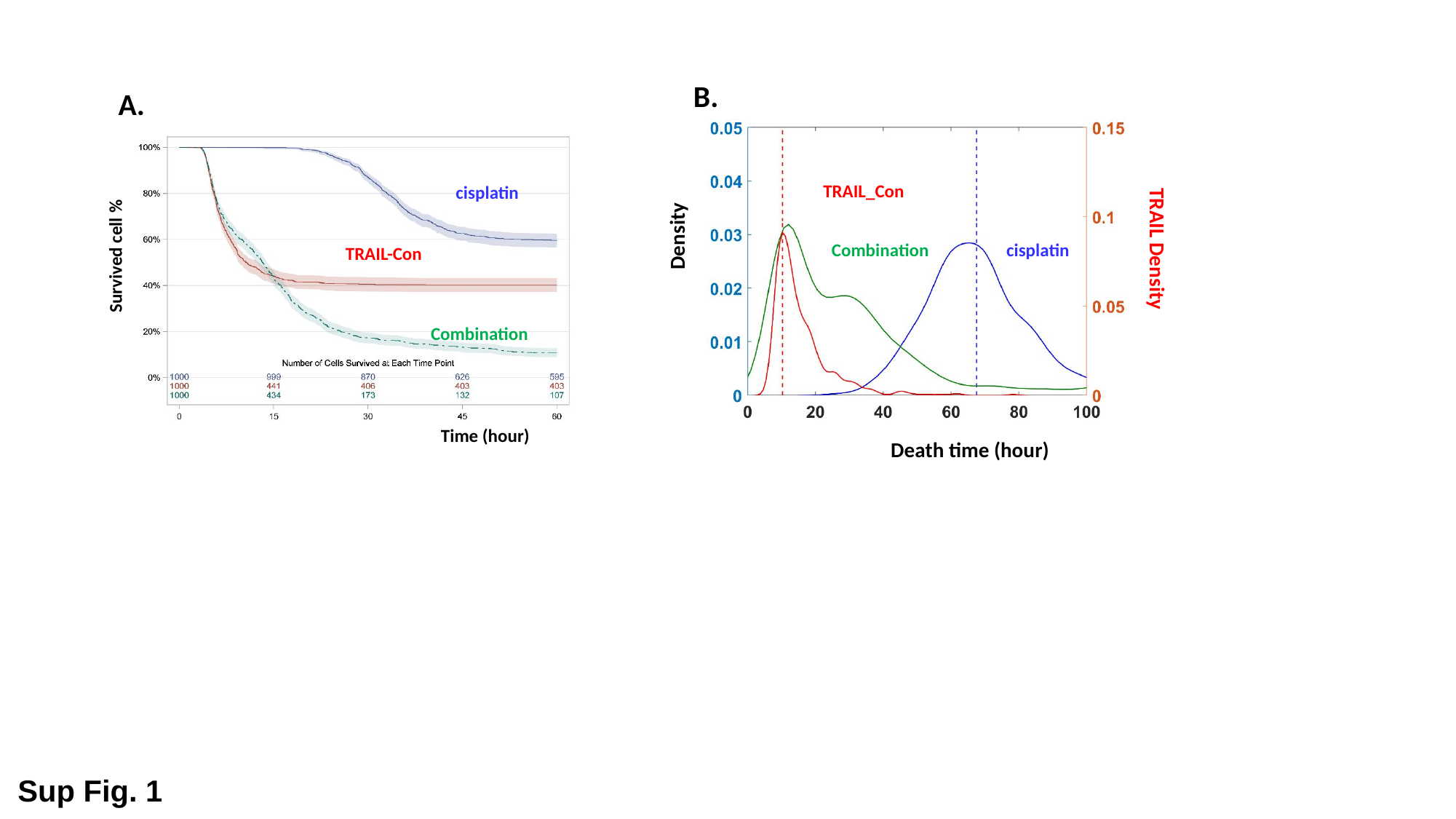

B.
A.
TRAIL_Con
cisplatin
Density
TRAIL Density
Combination
cisplatin
TRAIL-Con
Survived cell %
Combination
Time (hour)
Death time (hour)
Sup Fig. 1
