# Supplementary material for: Designing combination therapies with modeling chaperoned machine learning
Source: PLoS Comput Biol. 2019 Sep 9;15(9):e1007158. doi: 10.1371/journal.pcbi.1007158 (PMC6733436; doi:10.1371/journal.pcbi.1007158)
Supplement: S1 Data — (DOCX) [file pcbi.1007158.s005.docx]

**Supporting Data:** Single cell data. The data is separated into 2 different sheets. In each sheet, the rows correspond to a single tracked cell and the columns correspond to the data at a given timepoint (297 15 minute timepoints). The sheets are labeled (i) p53: p53-Venus levels. A ‘-1’ indicates that a cell was not tracked at that timepoint due to cell death. Note the TRAIL data does not include p53-Venus levels as we were unable to track these cells over time (ii) Apoptosis: A ‘1’ indicates the cell died at that time.
