# Supplementary material for: Designing combination therapies with modeling chaperoned machine learning
Source: PLoS Comput Biol. 2019 Sep 9;15(9):e1007158. doi: 10.1371/journal.pcbi.1007158 (PMC6733436; doi:10.1371/journal.pcbi.1007158)
Supplement: S1 Table — (DOCX) [file pcbi.1007158.s009.docx]

**Supplementary Table 1a. Equations of the model**

| BCL Family Module | |  |
| --- | --- | --- |
| Algebraic Equations | |  |
| $Bax=BaxT-BaxmT$ | $Baxm=BaxmT-Baxm:Bcl$ | |
| $Bh3=Bh3T-Bh3:Bcl$ | $Bcl2=Bcl2T-Baxm:Bcl-Bh3:Bcl$ | |
| Differential Equations | | |
| Total Mitochondrial Bax | | |
| $\frac{\boldsymbol{dBaxmT}}{\boldsymbol{dt}}\boldsymbol{=}\left( \boldsymbol{k}_{\boldsymbol{f}\boldsymbol{1}}\boldsymbol{+}\boldsymbol{k}_{\boldsymbol{f}\boldsymbol{2}}\boldsymbol{*Bh}\boldsymbol{3} \right)\boldsymbol{*Bax-}\boldsymbol{k}_{\boldsymbol{b}}\boldsymbol{*BaxmT}$ | |  |
| Mitochondrial Bax / Bcl2 Dimer | |  |
| $\frac{\boldsymbol{dBaxm:Bcl}\boldsymbol{2}}{\boldsymbol{dt}}\boldsymbol{=}\boldsymbol{k}_{\boldsymbol{asX}\boldsymbol{2}}\boldsymbol{*Baxm*Bcl}\boldsymbol{2-}\boldsymbol{k}_{\boldsymbol{dsX}\boldsymbol{2}}\boldsymbol{*Baxm:Bcl}\boldsymbol{2 -}\boldsymbol{k}_{\boldsymbol{b}}\boldsymbol{*Baxm:Bcl}\boldsymbol{2}$ | |  |
| Bh3 / Bcl2 Dimer | |  |
| $\frac{\boldsymbol{dBh}\boldsymbol{3:Bcl}\boldsymbol{2}}{\boldsymbol{dt}}\boldsymbol{=}\boldsymbol{k}_{\boldsymbol{as}\boldsymbol{32}}\boldsymbol{*Bh}\boldsymbol{3*Bcl}\boldsymbol{2-}\boldsymbol{k}_{\boldsymbol{ds}\boldsymbol{32}}\boldsymbol{*Bh}\boldsymbol{3:Bcl}\boldsymbol{2}$ | |  |
| Bh3 Total | |  |
| $\frac{\boldsymbol{Bh}\boldsymbol{3}\boldsymbol{T}}{\boldsymbol{dt}}\boldsymbol{=}\boldsymbol{k}_{\boldsymbol{sBh}\boldsymbol{3}}\boldsymbol{+}\boldsymbol{k}_{\boldsymbol{s}\boldsymbol{2}}\boldsymbol{*p}\boldsymbol{53+}\boldsymbol{k}_{\boldsymbol{s}\boldsymbol{3}}\boldsymbol{*Caspase}\boldsymbol{8+}\boldsymbol{k}_{\boldsymbol{s}\boldsymbol{4}}\boldsymbol{*DISC-}\boldsymbol{k}_{\boldsymbol{dBH}\boldsymbol{3}}\boldsymbol{*Bh}\boldsymbol{3}\boldsymbol{T}$ | |  |
|  | |  |
| DISC Module | |  |
| Caspase 8 | |  |
| $\frac{\boldsymbol{dCaspase}\boldsymbol{8}}{\boldsymbol{dt}}\boldsymbol{=}\boldsymbol{k}_{\boldsymbol{aC}\boldsymbol{8}}\boldsymbol{*Trail-}\left( \boldsymbol{k}_{\boldsymbol{iC}\boldsymbol{8}}\boldsymbol{+}\boldsymbol{k}_{\boldsymbol{i}\boldsymbol{2}}\boldsymbol{*cIAP} \right)\boldsymbol{*Caspase}\boldsymbol{8}$ | |  |
| Death-Induced Signaling Complex (DISC) | |  |
| $\frac{\boldsymbol{dDISC}}{\boldsymbol{dt}}\boldsymbol{=}\boldsymbol{k}_{\boldsymbol{aDISC}}\boldsymbol{-}\left( \boldsymbol{k}_{\boldsymbol{iDISC}}\boldsymbol{+}\boldsymbol{k}_{\boldsymbol{iD}\boldsymbol{2}}\boldsymbol{*cIAP} \right)\boldsymbol{*DISC}$ | |  |
| cIAP | |  |
| $\frac{\boldsymbol{dcIAP}}{\boldsymbol{dt}}\boldsymbol{=}\boldsymbol{k}_{\boldsymbol{sIAP}}\boldsymbol{+}\boldsymbol{k}_{\boldsymbol{sIAP}\boldsymbol{2}}\boldsymbol{*}\frac{\boldsymbol{Dru}\boldsymbol{g}^{\boldsymbol{n}}}{\boldsymbol{Dru}\boldsymbol{g}^{\boldsymbol{n}}\boldsymbol{+}\boldsymbol{J}^{\boldsymbol{n}}}\boldsymbol{-}\boldsymbol{k}_{\boldsymbol{dIAP}}\boldsymbol{*cIAP}$ | |  |
|  | |  |
| P53 Signaling Module | |  |
| Algebraic Equations | |  |
| $\boldsymbol{wp}\boldsymbol{53=}\boldsymbol{R}_{\boldsymbol{0}}^{\boldsymbol{p}\boldsymbol{53}}\boldsymbol{+}\boldsymbol{R}_{\boldsymbol{p}\boldsymbol{53}}^{\boldsymbol{p}\boldsymbol{53}}\boldsymbol{*p}\boldsymbol{53}$ *+*$\boldsymbol{R}_{\boldsymbol{Mdm}\boldsymbol{2}}^{\boldsymbol{p}\boldsymbol{53}}\boldsymbol{*Mdm}\boldsymbol{2}$ | $wMdm2=R_{0}^{Mdm2}+R_{p53}^{Mdm2}*p53$ |  |
| $\boldsymbol{Fp}\boldsymbol{53=}\frac{\boldsymbol{1}}{\boldsymbol{1+}\boldsymbol{e}^{\boldsymbol{- \sigma\cdot wp}\boldsymbol{53}}}$ | $FMdm2=\frac{1}{1+e^{- \sigma*Drug}}+\frac{1}{1+e^{- \sigma_{2} \cdot wMdm2}}$ |  |
| Differential Equations | |  |
| P53 | |  |
| $\frac{\boldsymbol{dp}\boldsymbol{53}}{\boldsymbol{dt}}\boldsymbol{=tsp}\boldsymbol{53*(Fp}\boldsymbol{53-p}\boldsymbol{53)}$ | |  |
| Mdm2 | |  |
| $\frac{\boldsymbol{dMdm}\boldsymbol{2}}{\boldsymbol{dt}}\boldsymbol{=tsMdm}\boldsymbol{2*(FMdm}\boldsymbol{2-Mdm}\boldsymbol{2)}$ | |  |
|  | |  |
| Caspase Signaling Module | |  |
| Algebraic Equations | |  |
| $\boldsymbol{smac=smaccyto-smac:xiap}$ | $xiap=xiapT-smac:xiap-c3:xiap-c9:xiap$ |  |
| $\boldsymbol{Baxm=Bax*100}$ |  |  |
| Differential Equations | |  |
| Channels Opened on Mitochondria | |  |
| $\frac{\boldsymbol{dCO}}{\boldsymbol{dt}}\boldsymbol{=}\boldsymbol{k}_{\boldsymbol{open}}\boldsymbol{*}\boldsymbol{Baxm}^{\boldsymbol{m}}\boldsymbol{*}\left( \boldsymbol{1-CO} \right)\boldsymbol{-}\boldsymbol{k}_{\boldsymbol{close}}\boldsymbol{*CO}$ | |  |
| Smac in Mitochondria | |  |
| $\frac{\boldsymbol{dsmacmito}}{\boldsymbol{dt}}\boldsymbol{=-CO*smacmito}$ | |  |
| Cytochrome C in Mitochondria | |  |
| $\frac{\boldsymbol{dcytocmito}}{\boldsymbol{dt}}\boldsymbol{=-CO*cytocmito}$ | |  |
| Cytochrome c in cytoplasm | |  |
| $\frac{\boldsymbol{dcytoc}}{\boldsymbol{dt}}\boldsymbol{=0.01*CO*cytocmito-}\boldsymbol{k}_{\boldsymbol{dcytoc}}\boldsymbol{*cytoc}$ | |  |
| Smac in Cytoplasm | |  |
| $\frac{\boldsymbol{dsmaccyto}}{\boldsymbol{dt}}\boldsymbol{=0.01*CO*smacmito-}\boldsymbol{k}_{\boldsymbol{dsmaccyto}}\boldsymbol{*smac-}\boldsymbol{k}_{\boldsymbol{dsx}}\boldsymbol{*smac:xiap}$ | |  |
| Procaspase 3 | |  |
| $\frac{\boldsymbol{dproc}\boldsymbol{3}}{\boldsymbol{dt}}\boldsymbol{=}\boldsymbol{k}_{\boldsymbol{sproc}\boldsymbol{3}}\boldsymbol{-}\boldsymbol{k}_{\boldsymbol{dproc}\boldsymbol{3}}\boldsymbol{*proc}\boldsymbol{3-}\boldsymbol{(k}_{\boldsymbol{ac}\boldsymbol{3}\boldsymbol{bk}}\boldsymbol{+}\boldsymbol{k}_{\boldsymbol{ac}\boldsymbol{3}}^{\boldsymbol{''}}\boldsymbol{*}{\boldsymbol{c}\boldsymbol{9}}^{\boldsymbol{n}}\boldsymbol{+}\boldsymbol{k}_{\boldsymbol{ac}\boldsymbol{3}}^{\boldsymbol{'''}}\boldsymbol{*a}{\boldsymbol{c}\boldsymbol{9}}^{\boldsymbol{n}}\boldsymbol{+}\boldsymbol{k}_{\boldsymbol{ac}\boldsymbol{3}\boldsymbol{by}\boldsymbol{8}}\boldsymbol{*caspase}\boldsymbol{8)*proc}\boldsymbol{3}$ | |  |
| Procaspase 9 | |  |
| $\frac{\boldsymbol{dproc}\boldsymbol{9}}{\boldsymbol{dt}}\boldsymbol{=}\boldsymbol{k}_{\boldsymbol{sproc}\boldsymbol{9}}\boldsymbol{-}\boldsymbol{k}_{\boldsymbol{dproc}\boldsymbol{9}}\boldsymbol{*proc}\boldsymbol{9-}\boldsymbol{k}_{\boldsymbol{sc}\boldsymbol{9}}\boldsymbol{*proc}\boldsymbol{9*}\boldsymbol{cytoc}^{\boldsymbol{n}}$ | |  |
| Caspase9 | |  |
| $\frac{\boldsymbol{dc}\boldsymbol{9}}{\boldsymbol{dt}}\boldsymbol{=}\boldsymbol{k}_{\boldsymbol{sc}\boldsymbol{9}}\boldsymbol{*proc}\boldsymbol{9*}\boldsymbol{cytoc}^{\boldsymbol{n}}\boldsymbol{-}\boldsymbol{k}_{\boldsymbol{dc}\boldsymbol{9}}\boldsymbol{*c}\boldsymbol{9-}\left( \boldsymbol{k}_{\boldsymbol{ac}\boldsymbol{9}}^{\boldsymbol{'}}\boldsymbol{+}\boldsymbol{k}_{\boldsymbol{ac}\boldsymbol{9}}^{\boldsymbol{''}}\boldsymbol{*}{\boldsymbol{c}\boldsymbol{3}}^{\boldsymbol{n}} \right)\boldsymbol{*c}\boldsymbol{9-}\boldsymbol{k}_{\boldsymbol{as}\boldsymbol{9}\boldsymbol{x}}\boldsymbol{*c}\boldsymbol{9*xiap+}\boldsymbol{k}_{\boldsymbol{ds}\boldsymbol{9}\boldsymbol{x}}\boldsymbol{*c}\boldsymbol{9:xiap}$ | |  |
| Caspase 9 / Xiap complex | |  |
| $\frac{\boldsymbol{dc}\boldsymbol{9:xiap}}{\boldsymbol{dt}}\boldsymbol{=}\boldsymbol{k}_{\boldsymbol{as}\boldsymbol{9}\boldsymbol{x}}\boldsymbol{*c}\boldsymbol{9}\boldsymbol{*xiap-}\boldsymbol{k}_{\boldsymbol{ds}\boldsymbol{9}\boldsymbol{x}}\boldsymbol{*c}\boldsymbol{9:xiap-}\boldsymbol{k}_{\boldsymbol{d}\boldsymbol{9}\boldsymbol{x}}\boldsymbol{*c}\boldsymbol{9:xiap}$ | |  |
| Activated Caspase 9 | |  |
| $\frac{\boldsymbol{dac}\boldsymbol{9}}{\boldsymbol{dt}}\boldsymbol{=}\boldsymbol{(k}_{\boldsymbol{ac}\boldsymbol{9}}^{\boldsymbol{'}}\boldsymbol{+}\boldsymbol{k}_{\boldsymbol{ac}\boldsymbol{9}}^{\boldsymbol{''}}\boldsymbol{*}{\boldsymbol{c}\boldsymbol{3}}^{\boldsymbol{n}}\boldsymbol{)*c}\boldsymbol{9}\boldsymbol{-}\boldsymbol{k}_{\boldsymbol{dac}\boldsymbol{9}}\boldsymbol{*ac}\boldsymbol{9}$ | |  |
| Caspase 3 | |  |
| $\frac{\boldsymbol{dc}\boldsymbol{3}}{\boldsymbol{dt}}\boldsymbol{=}\left( \boldsymbol{k}_{\boldsymbol{ac}\boldsymbol{3}\boldsymbol{bk}}\boldsymbol{+}\boldsymbol{k}_{\boldsymbol{ac}\boldsymbol{3}}^{\boldsymbol{''}}\boldsymbol{*}{\boldsymbol{c}\boldsymbol{9}}^{\boldsymbol{n}}\boldsymbol{+}\boldsymbol{k}_{\boldsymbol{ac}\boldsymbol{3}}^{\boldsymbol{'''}}\boldsymbol{*}{\boldsymbol{ac}\boldsymbol{9}}^{\boldsymbol{n}}\boldsymbol{+}\boldsymbol{k}_{\boldsymbol{ac}\boldsymbol{3}\boldsymbol{by}\boldsymbol{8}}\boldsymbol{*caspase}\boldsymbol{8} \right)\boldsymbol{*proc}\boldsymbol{3}\boldsymbol{-}\boldsymbol{k}_{\boldsymbol{dc}\boldsymbol{3}}\boldsymbol{*c}\boldsymbol{3-}\boldsymbol{k}_{\boldsymbol{as}\boldsymbol{3}\boldsymbol{x}}\boldsymbol{*c}\boldsymbol{3*xiap+}\boldsymbol{k}_{\boldsymbol{ds}\boldsymbol{3}\boldsymbol{x}}\boldsymbol{*c}\boldsymbol{3:xiap}$ | |  |
| Caspase 3 / Xiap complex | |  |
| $\frac{\boldsymbol{dc}\boldsymbol{3:xiap}}{\boldsymbol{dt}}\boldsymbol{=}\boldsymbol{k}_{\boldsymbol{as}\boldsymbol{3}\boldsymbol{x}}\boldsymbol{*c}\boldsymbol{3*xiap-}\boldsymbol{k}_{\boldsymbol{ds}\boldsymbol{3}\boldsymbol{x}}\boldsymbol{*c}\boldsymbol{3:xiap-}\boldsymbol{k}_{\boldsymbol{d}\boldsymbol{3}\boldsymbol{x}}\boldsymbol{*c}\boldsymbol{3:xiap}$ | |  |
| Smac / Xiap complex | |  |
| $\frac{\boldsymbol{dsmac:xiap}}{\boldsymbol{dt}}\boldsymbol{=}\boldsymbol{k}_{\boldsymbol{assx}}\boldsymbol{*smac*xiap-}\boldsymbol{k}_{\boldsymbol{dssx}}\boldsymbol{*smac:xiap-}\boldsymbol{k}_{\boldsymbol{dsx}}\boldsymbol{*smac:xiap}$ | |  |

**Table 1b. Parameters and initial conditions of the model**

| Parameters | | | | | |
| --- | --- | --- | --- | --- | --- |
| Background rate by which Bax translocate to mitochondria | | Bh3 enhanced rate of translocation of Bax to mitochondria | | Rate by which Mitochondrial Bax translocate back to cytoplasm | Rate of association of Baxm:Bcl2 Dimer |
| $\boldsymbol{k}_{\boldsymbol{f}\boldsymbol{1}}\boldsymbol{=1}$ | | $k_{f2}=300$ | | $k_{b}=2$ | $k_{asx2}=9000$ |
| Rate of Dissociation of Baxm:Bcl2 Dimer | | Rate of Bh3:Bcl2 Dimer association | | Rate of Dissociation of Bh3:Bcl2 Dimer | Total Bax |
| $\boldsymbol{k}_{\boldsymbol{dsx}\boldsymbol{2}}\boldsymbol{=0.05}$ | | $k_{as32}=1000$ | | $k_{ds32}=0.01$ | $BaxT=1$ |
| Total Bcl2 | Background Production Rate of Bh3 | | p53 enhanced rate of Bh3 production | | Caspase 8 enhanced rate of Bh3 production |
| $\boldsymbol{Bcl}\boldsymbol{2}\boldsymbol{T=0.8}$ | $k_{sBh3}=0.1$ | | $k_{s2}=0.2$ | | $k_{s3}=1$ |
| DISC enhanced rate of Bh3 Production | | Rate of Bh3 Degradation | | Rate of Caspase 8 Activation by TRAIL | Inactivation rate of Caspase8 |
| $\boldsymbol{k}_{\boldsymbol{s}\boldsymbol{4}}\boldsymbol{=1}$ | | $k_{dBh3}=1$ | | $k_{aC8}=0.01$ | $k_{iC8}=0.1$ |
| Rate of cIAP enhanced Caspase8 inactivation | | Hill constant for cIAP production by Cisplatin | | Background production rate of cIAP | Cisplatin enhanced production of cIAP |
| $\boldsymbol{k}_{\boldsymbol{i}\boldsymbol{2}}\boldsymbol{=0.15}$ | | $J=0.1$ | | $k_{sIAP}=0.001$ | $k_{sIAP2}=0.04$ |
| Degradation rate of cIAP | | Background activation rate of DISC | | Inhibition rate of DISC | Inhibition of DISC due to cIAP |
| $\boldsymbol{k}_{\boldsymbol{dIAP}}\boldsymbol{=0.01}$ | | $k_{aDISC}=0.01$ | | $k_{iDISC}=0.1$ | $k_{iD2}=0.15$ |
| Background activation of p53 | | Self- Activation of p53 | | Background activation of Mdm2 | Activation of Mdm2 due to p53 |
| $\boldsymbol{R}_{\boldsymbol{0}}^{\boldsymbol{p}\boldsymbol{53}}\boldsymbol{= -0.4}$ | | $R_{p53}^{p53}=2$ | | $R_{0}^{Mdm2}=-0.55$ | $R_{p53}^{Mdm2}=1$ |
| Inhibition of p53 due to Mdm2 | | Time scale of p53 | | Time scale of Mdm2 | Concentration of Cisplatin |
| $\boldsymbol{R}_{\boldsymbol{Mdm2}}^{\boldsymbol{p}\boldsymbol{53}}\boldsymbol{=-1}$ | | $tsp53=0.24$ | | $tsMdm2=0.12$ | $drug=0.45$ |
| Non-linearity factors | | Total amount of Xiap in system | | Background activation of Caspase 3 | Background production of procaspase 9 |
| $\boldsymbol{\sigma=5}$,$\boldsymbol{\sigma}_{\boldsymbol{2}}\boldsymbol{=10}$ | | $xiapT=6$ | | $k_{ac3bk}=0.001$ | $k_{sproc9}=0.001$ |
| Degradation rate of procaspase9 | | Background activation rate of caspase 9 | | Rate of Mitochondrial channel opening | Number of Baxm molecules for the mitochondria channel |
| $\boldsymbol{k}_{\boldsymbol{dproc}\boldsymbol{9}}\boldsymbol{=0.001}$ | | $k_{ac9}^{'}=0.001$ | | $k_{open}=10$ | $m=4$ |
| Rate of Mitochondrial channel closure | | Activation rate of Caspase 9 due to Caspase 3 | | Degradation rate of Cytochrome C | Degradation rate of Cytoplasmic Smac |
| $\boldsymbol{k}_{\boldsymbol{close}}\mathbf{=1000}$ | | $k_{ac9}^{''}=0.5$ | | $k_{dcytoc}=0.005$ | $k_{dsmaccyto}=0.0001$ |
| Background production rate of procaspase 3 | | Degradation rate of procaspase3 | | Caspase 9 activation due to cytochrome c | Hill coefficient |
| $\boldsymbol{k}_{\boldsymbol{sproc}\boldsymbol{3}}\boldsymbol{=0.002}$ | | $k_{dproc3}=0.001$ | | $k_{sc9}=0.001$ | $n=2$ |
| Degradation rate of caspase 9 | | Degradation rate of active caspase 9 | | Activation rate of c3 due to Caspase 9 | Activation rate of c3 due to active Caspase 9 |
| $\boldsymbol{k}_{\boldsymbol{dc}\boldsymbol{9}}\boldsymbol{=0.002}$ | | $k_{dac9}=0.003$ | | $k_{ac3}^{''}=0.02$ | $k_{ac3}^{'''}=0.5$ |
| Activation of Caspase 3 by caspase8 | | Degradation rate of Caspase3 | | Association rate of caspase3:xiap complex | Dissociation rate of caspase3:xiap complex |
| $\boldsymbol{k}_{\boldsymbol{ac}\boldsymbol{3}\boldsymbol{by}\boldsymbol{8}}\boldsymbol{=0.01}$ | | $k_{dc3}=0.002$ | | $k_{as3x}=0.2$ | $k_{ds3x}=0.5$ |
| Degradation rate of caspase3:xiap complex | | Association rate of caspase9:xiap complex | | Dissociation rate of caspase9:xiap complex | Degradation rate of caspase9:xiap complex |
| $\boldsymbol{k}_{\boldsymbol{d}\boldsymbol{3}\boldsymbol{x}}\boldsymbol{=0.1}$ | | $k_{as9x}=0.1$ | | $k_{ds9x}=0.6$ | $k_{d9x}=0.2$ |
| Association rate of Smac:xiap complex | | Dissociation rate of Smac:xiap complex | | Degradation rate of Smac:xiap complex |  |
| $\boldsymbol{k}_{\boldsymbol{assx}}\boldsymbol{=2}$ | | $k_{dssx}=0.01$ | | $k_{dsx}=0.007$ |  |
| Initial Conditions | | | | | |
| $\boldsymbol{BaxmT=0.3}$ | | $Baxm:Bcl=0.33$ | | $Bh3:Bcl=0.16$ | $P53=0$ |
| $\boldsymbol{Mdm}\boldsymbol{2=1}$ | | $CIAP=0$ | | $cytoc=0.1$ | $DISC=0$ |
| $\boldsymbol{c}\boldsymbol{9=0}$ | | $ac9=0$ | | $c3=0$ |  |
